# Quality of life of students of a private medical college

**DOI:** 10.12669/pjms.36.2.668

**Published:** 2020

**Authors:** Yasir Aziz, Ahmad Yar Khan, Iqra Shahid, Muhammad Athar Khan, Aisha

**Affiliations:** 1 Yasir Aziz, Final Year Medical Students Liaquat College of Medicine & Dentistry, Karachi, Pakistan; 2 Ahmad Yar Khan, Final Year Medical Students Liaquat College of Medicine & Dentistry, Karachi, Pakistan; 3 Iqra Shahid, Final Year Medical Students Liaquat College of Medicine & Dentistry, Karachi, Pakistan; 4 Muhammad Athar Khan, Associate Professor, Department of Community Medicine, Liaquat College of Medicine & Dentistry, Karachi, Pakistan; 5 Aisha, Private General Practitioner, Ex-Student Liaquat College of Medicine & Dentistry, Liaquat College of Medicine & Dentistry, Karachi, Pakistan

**Keywords:** Medical education, Medical student, Quality of life, Academic load

## Abstract

**Objective:**

To determine the quality of life of students of a private medical college in Karachi in Pakistan.

**Methods:**

This cross sectional study was conducted among 217 medical students of Liaquat College of Medicine and Dentistry, Karachi from June 2017 to March 2018. Students were selected by a stratified sampling method and the World Health Organization Quality of Life BREF Instruments (WHO QOL-BREF) was used for the above-mentioned study. Statistical analysis was performed using SPSS (Statistical Package for Social Sciences) version 21 and Analysis of variance (ANOVA). Independent t-test was used as p <0.05 significant.

**Results:**

A total of 250 questionnaires were distributed among 2^nd^ year, 3^rd^ year, 4^th^ year and final year students and the response rate was 86.8%. Among them 48.5% (n=105) students were male and 51.5% (n=112) students were female, while 9.2% (n=20) students were currently ill and the other 90.8% (n=197) were healthy.

**Conclusion:**

Medical education influences the quality of life (QOL) of students adversely. Social relationships and environmental domain were satisfactory in private medical institutes whereas physical and psychological progress was low due to academic load which requires improvement either by physical activities such as fitness classes or other extra-curricular activities.

## INTRODUCTION

According to World Health Organization Quality of Life (WHO QOL) is defined as, “An individual’s perception of their position in life in the context of culture and value system in which they live, and in relation of their goals, expectations, standards and concerns”.^1^ Quality of Life (QOL) is the broad context encompassing Health Related Quality of life(HRQOL).^2^ As we know that the World Health Organization (WHO) have explained the quality of life, but they weren’t able to determine the minimum level of quality of life, considering the age, gender, occupation, and culture.^3^

Obtaining admission in medical college these days is a challenge for all the students. However, once acquired, the students further have to face academic challenges within the institution itself. This eventually brings them in a state of stress and depression which is a risk for cardiovascular diseases and anxiety disordes. Medical students adapt poor lifestyle and unhealthy habits such as disturbed sleep cycles, irregular meal timings, smoking, and increased nicotine and caffeine use.^4^ As a result, maintaining some balance between their academic performance and quality of life becomes a difficult task. Research shows that increased level of stress and suffering from depression for a longer period of time will have poor effect on the attitude, personality, learning abilities and academic performance of the medical students eventually resulting in poor career performance and lower quality of patient care in future.^1^ Puthran R’s analysis of a study showed that stress and depression rate was much higher in the beginning of medical profession as compared to the senior students. The reason was that the newer students had to make adjustments to their newer lifestyle. However, the situation later improves because they learn to cope with their issues that they had to face in the initial time.^5^ Medical students suffer from psychological stress not just due to high study load, but also because they have narrow professional employment opportunities.^6^

According to studies young people in general population have high quality of life as compared to the quality of life of young medical students.^7^ The occurrence of stress and anxiety among medical students was highly prevalent according to the systemic review of 40 studies.^8^ According to studies it was calculated that depression and anxiety will be the second most common cause of disability worldwide.^9^ Quality of life assessment instrument of World Health Organization Quality of Life (WHO QOL-BREF) was an internationally acceptable instrument.

In this study, we aimed to investigate two things. One was to understand how the biological, psychological, socio-economic factors, as well as, age and gender, influence the health of medical students and second was to elaborate the perception of students about their quality of life and its relationship with medical education. The researchers also wanted to know how social variables related to health and medical course simultaneously influence the quality of life. The objective of this study was to determine the quality of life in medical students of a private medical college, Karachi by using WHO QOL-BREF.

## METHODS

The study design of this research was cross sectional and the sampling method was non-probability convenience sampling. The study was conducted on medical students of Liaquat College of Medicine and Dentistry, Karachi from June 2017 to March 2018. We included students of 2^nd^, 3^rd^, 4^th^ and final year. We calculated our sample size by the help of Cochran’s formula. Sample size of the study was 217 out of which 71 students were from 2^nd^ year, 48 from 3^rd^ year, 65 from 4^th^ year and 33 were from the final year. We started from 2^nd^ year because they had recently passed through the initial difficulties of medical field. We compared our study between male and female students with no age limits. Similarly, no ethnic or racial differences were considered. However, 1^st^ year students and students of dental departments were excluded. WHO QOL-BREF questionnaire were used during the service. Furthermore, only completed questionnaires were included for analysis which was done using SPSS 21. Basic variables were analyzed using descriptive statistics for finding frequencies and percentages, mean and standard deviation of QOL and its domains. SPSS syntax version given in WHO QOL-BREF manual was followed and used to calculate the domain scores. Raw domain scores were then transformed to a 4-20 score, according to the guidelines. The mean score of items within each domain was used to calculate the domain score. These scores were then transformed linearly to a 100-scale using the below formula, with 100 being the most favorable score, and 0 being the least favorable score.^1^

Transformed score= (Score-4) X (100/16).

### Statistical analysis:

Statistical analysis was performed using SPSS (Statistical Package for Social Sciences) version 21. Percentages (frequencies) were used for qualitative variables and mean±sd was referred to for quantitative variables. Analysis of Variance (ANOVA) and Independent t-test was performed for comparison as p <0.05 significant.

## RESULTS

In the present study total of 250 questionnaire were distributed among the students of 2^nd^ year, 3^rd^ year, 4^th^ year and final year. After collecting the questionnaire from all 250 students, the analysis continued. Thirty three students did not respond and thus, according to WHO QOL-BREF instruction manual, were excluded. These 33 missing questionnaires from the data, therefore, reduced the response rate to 86.8%. Among these volunteer participants, 48.5% (n=105) students were male and 51.6% (n=112) students were female. The maximum number of students who participated were from 2^nd^ year; 32.7% (n=71), 22.1% (n=48) students were from 3^rd^ year 30% (n=65) of students were from 4^th^ year and 15.3% (n=33) of students were from final year. 4.1% (n=9) were married and 95.1% (n=208) students were single. Among all only 9.2% (n=20) were ill during the conduction period of study and the remaining 90.8% (n=197) were healthy Table-I.

**Table-I:** Baseline characteristics of study participants (n=217).

Characteristics		n	%
Gender	Male	105	48.4
	Female	112	51.6
Year of MBBS	2^nd^ Year	71	32.7
	3^rd^ Year	48	22.1
	4^th^ Year	65	30.0
	5^th^ Year	33	15.2
Marital Status	Single	208	95.9
	Married	9	4.1
Current Illness	Yes	20	9.2
	No	197	90.8

The overall mean score of QOL was 64.7±17.6 while the mean score in physical domain was 62±14.9, psychological domain 64.5±17.3, social domain 66.1±20.7 and environmental domain 66.3±17.5. Table-II We found no statistically significant (p = 0.517) difference in QOL mean scores in different domains. No statistically significant difference in the comparison of mean scores of different domains with the year of study. Table-III

**Table II:** Mean differences of QOL and its domains (n=217).

Domain	Mean ± SD	p-value
General QOL	64.7±17.6	NS
Physical health	62±14.9	
Psychological	64.5±17.3	
Social relationship	66.1±20.7	
Environmental	66.3±17.5	

**Table-III:** Comparison of mean scores of different domains with year of study (n=217).

Domain	Year of Study				

	2^nd^ Mean±SD	3^rd^ Mean±SD	4^th^ Mean±SD	5^th^ Mean±SD	p-value
Physical health	61.33±14.55	59.66±14.85	64.04±16.18	62.72±13.16	NS
Psychological	63.50±16.34	66.31±19.01	61.84±19.17	69.42±11.14	NS
Social relationship	63.15±23.42	68.43±19.60	67.44±18.53	66.18±20.23	NS
Environmental	65.21±17.50	66.91±17.85	67.33±18.96	65.87±14.18	NS

**Fig.1:**
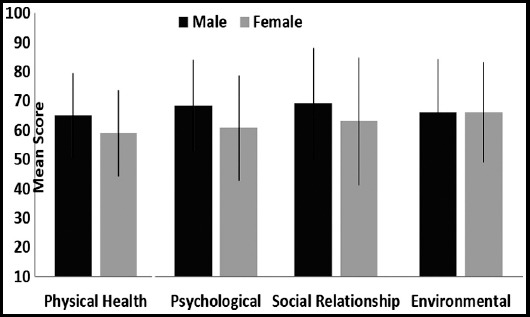
Comparison of gender with mean score of different domains (n = 217).

The mean comparison of different domain scores between male and female students was found insignificant, physical health (p=0.839), psychological health (p=0.153), social relationship (p=0.140) and environmental domain (p=0.648) Fig.1.

## DISCUSSION

The research conducted by WHO QOL team had physical domain of 70.4, physiological domain of 67.1, social domain of 67.7 and environmental domain of about 63.1 percent on quality of life of well study group.^10^ According to this pilot study conducted by WHO and a study conducted in Liaquat college of medicine and dentistry, Karachi had weak physical health domain of 61.99% with a 95% confidence interval (59.99-63.98). This domain was overall minimum in 3^rd^ MBBS students which was 59.67% with confidence interval of 95% (55.35-63.98). This showed decrease in QOL of 3^rd^ year students because of their first time exposure to the clinical wards of the hospital due to permanent presence of pressure and emotional stress in the hospital also shown by the study in Peshawar and India.^11^,^12^ Sleep deprivation was the main cause of the above-mentioned budding issue because medical students do not give prior importance to sleep because of academic necessities. They minimize their sleep hours in order to extend the working hours to cope with stressful workload. Studies have shown that poor coping strategies to manage academic load and burden is also one of the main stress factors in medical students.^13^ In the study “Quality of Life of Medical Students in China: Zhang Y et al. reported that the Quality of Life (QOL) of male medical students in different domains were; physical domain 68.59%, psychological domain 65.16%, social domain 62.75%, environmental domain 54.89%, and in female medical students physical domain 66.93%, psychological domain 63.55%, social domain 63.91%, environmental domain 54.96%. In comparison to the study conducted in China, the study of Liaquat College of Medicine and Dentistry, Karachi showed that the three domains i.e. psychological, social relationship and environmental domains are much better in males than study conducted in China.^6^

In this study, males had physical domain of 65.15%, psychological domain 68.48%, social relationship domain 69.19%, and environmental domain was 66.35%, and in female’s physical domain was 59.02%, psychological 60.82%, social relationship 63.14%, and environmental domain was 66.30%. To obtain a sustainable solution of problems, the coming generation should be provided with exact environmental knowledge and skills which are affected by environmental factors arising from the recent action. Thus, by improving the pro-environmental behaviour and their solution we will be able to raise a responsible, well-mannered and competent individual with better knowledge and skill values who will participate in developing the world with environmental sustainability.^14^ As long as our environmental domain was concerned, it was good in comparison to the WHO QOL-BREF. There were various reasons for the said result. Our study was conducted in private institute where people were mostly coming from the upper middle class. Moreover, because our institute has maintained the highest standards of discipline and respect, the student and staff ratio is on average, and the staff is able to maintain a student-friendly learning environment. On the other hand, the study conducted in Andhra Medical College, Visakhapatnam had the physical domain of 71.35%, psychological domain of 63.3%, social relationship domain being 69.55%, and environmental domain of 67.65.^15^ This, in comparison to the study conducted in Liaquat College of Medicine and Dentistry, Karachi whose physical domain was 61.99%, psychological domain 64.53%, social relationship domain 66.06%, and environmental domain 66.32%, were better in all physical, social relationship and environmental domains except the psychological domain which was better in Liaquat College of Medicine and Dentistry, Karachi. There was a wide range of variation in the physical domain between the Andhra Medical College, Visakhapatnam and Liaquat College of Medicine and Dentistry, Karachi. As challenges provided during study in the medical school program makes it difficult for a student to maintain healthy behavior and attitude over the full course of five years with adverse effects specifically on their physical health. In the medical school, workload affects the physical activity, diet quality and general health in bad manners. There is a great impact of passive self-assurance and student’s own interest over the career development of each student. In the field of study, those who show great interest in their studies are sincerer towards their academic performance, which yield better effects on QOL especially high appreciation of their physical and mental aspect. Those students who showed poor interest in their studies got stressed easily. The study conducted in Shifa International Islamabad, showed that the physical domain was 69.39%, psychological domain was 66.48%, social relationship domain was 68.68%, and environmental domain 70.43%, which as compared to the study in Liaquat College of Medicine and Dentistry, Karachi gave the physical domain of 61.99%, psychological domain 64.53%, social domain 66.06%, and environmental domain 66.32%. There was not much variation in the three domain psychological domain, social and environmental except the physical domain which had a wider variation.^16^

There is a strong relationship between the physical and psychological domain. Physical domain has the positive control on psychiatric i.e. depression, mood swings, nervousness, and apprehension. Medical studies and all its activities are sedentary, medical education require long hours of study which make student vulnerable to adapt sedentary behavior. All these findings result in poor physical quality of life. Studies had demonstrated that those who regularly participate in a fitness program or other exercise regimes on their own have increased physical and emotional quality of life. Poor physical and psychological quality of life always manifests itself as anxiety disorder.^17^ About five to 20% of the frequency in stress and depression among adults is due to mental health problems. Stress and depression not only effects the morals, focus, learning abilities and skill of adults but also is one of the major causes of suicidal attempt.^18^

### Limitations of the study

A single center and small sample size study so the results could not be generalizable. A multicenter and large sample size study should be conducted to assess the quality of life in medical students.

## CONCLUSION

Medical education greatly affects the quality of life of students mainly in physical and psychological domains. Social relationship domain and environmental domain were satisfactory in private institutes. Due to lack of time for recreation, long on-duty assignments, student abuse, and exposure to human suffering is an additional source of stress during medical studies. So for the improvement of quality of life of medical students fitness classes and extra-curricular activities should be arranged to increase the quality of life in all four domains i.e. physical, social relationship, psychological and environmental.

### Authors’ Contribution:

**YA, MAK, AYK and IS** conceived, designed & editing of the manuscript, are responsible for integrity of research.

**AYK, IS and A** did data collection and manuscript writing.

**YA, AYK and MAK** did review and final approval of manuscript.

**MAK** did the statistical analysis.
